# Extra-role interpersonal interactions in Chinese organizational contexts: construct, measurement, and validity

**DOI:** 10.3389/fpsyg.2025.1597133

**Published:** 2026-01-09

**Authors:** Xuemei Liu, Xiuwen Zhao, Lin Bai, Xue Qian

**Affiliations:** 1 School of Management, Xihua University, Chengdu, China; 2 Research Institute of International Economics and Management, Xihua University, Chengdu, China; 3 West Campus Office, Industry and Trade College, Chengdu, China

**Keywords:** Chinese organizational context, extra-role interpersonal interaction, *guanxi*, scale, validation

## Abstract

As organizational scholars have become critically attuned to management situations in different cultural backgrounds, interest in relevant interactions with work roles in the context of Chinese organizations has grown rapidly. However, no formal description of such scenarios has been proposed, nor has a valid scale to measure extra-role interpersonal interaction (ERII) in Chinese organizational contexts been developed. To address this, we conducted a systematic multi-study-scale development project. We developed a 12-item measure of ERII comprising three dimensions: interaction for belonging, recreational interaction, and emotional interaction, and used this measure to empirically test the correlation and discriminant validity of ERII and related concept scales. In Study 1, Grounded Theory was used to explore the connotations and dimensions of ERII through interviews and coding, and an initial 19-item scale was formed. In Study 2, we conducted an exploratory factor analysis using data collected from organizations with 359 samples from collaborative backgrounds and online platforms to test and revise the initial scale. In Study 3, we performed confirmatory factor analysis with an additional sample of 715 to confirm that the 3-dimensional and 12-item ERII scale supported the qualitative study coding conclusions. In Study 4, we conducted criterion and discriminant validity tests using 228 samples collected online to prove the validity and discriminability of the ERII construct and the measurement. Collectively, the four studies provide preliminary evidence for the reliability and validity of the ERII scale, as well as for a theoretical model linking ERII to meaningful individual and organizational outcomes.

## Introduction

Working relationships with one’s line leader and work team partners are important interaction foundation that result in interpersonal understanding (Schermuly and Meyer, 2016; Banks et al., 2014; Hawkes and Neale, 2020; Methot et al., 2021). Most of these interactions are aimed at completing work tasks and can include discussing problems at work and negotiating solutions (Mikkola and Valkonen, 2019). In addition, employees may communicate through hobbies and express emotions with other members or groups in their organization or team after work or during breaks (van Zoonen et al., 2020). Such interactions can take place in a variety of settings, including work lounges, elevators, during commutes, or in outdoor spaces. Although existing literature examines some of these non-task-oriented, discretionary interactions (Chou et al., 2006; Minchella et al., 2023; Oh et al., 2004; Winslow et al., 2019), the specific focus on interactions occurring beyond the formal job responsibilities—particularly in Chinese organizations—remains underexplored.

In Chinese organizations, the most meaningful interpersonal interactions are typically based on job role relationships rather than job requirements or task orientation (Kang et al., 2024; Nolan and Rowley, 2020). Team members may increase their social relations or obligations to each other through these interactions, promoting the establishment of stable and positive interpersonal relationships and improving the performance of their job responsibilities (Lin and Kwantes, 2015; Dimotakis et al., 2011). However, managers and researchers have tended to pay more attention to functional interactions under the constraints of formal institutions or norms rather than spontaneous, non-work-oriented interpersonal interactions. Thus, the definition of the latter is not clear (Sinha and Singh-Sengupta, 1991; Lion and Gruenfeld, 1993) and it is difficult to explain employees and teams’ behaviors in the context of Chinese *guanxi* culture (He et al., 2024; Zhang et al., 2015). *Guanxi* is a complex adaptive system formed by strategically establishing, evolving, utilizing, and maintaining personal relationships based on social norms of trust and reciprocal obligation unique to the Chinese culture (Tsui and Farh, 1997; Chang et al., 2014). While *guanxi* often encompasses instrumental, reciprocal, and long-term obligations within a personalized network (Luo, 2011), the interpersonal interactions rooted in work roles that we focus on are distinct. They are characterized as rapidly established interactions based on shared professional identities (e.g., colleagues and superiors-subordinates), which are marked by weaker tie strength, more uncertain emotional commitment, and a less determinate instrumental focus compared to traditional *guanxi* (Park and Luo, 2001; Peng and Luo, 2000; Chen et al., 2013).

Indeed, substantial research has been conducted on interactions arising from work roles and role transitions, as well as the interplay between work and non-work domains (Liang, 2018; Pillemer and Rothbard, 2018; Yang and Wong, 2020), underscoring the need to precisely define such phenomena in the Chinese context. Although technological transformation has spurred organizational evolution, the core of such shifts lies in the dynamic and high-quality consensus and collaboration among team members, which arises from role-based interpersonal interactions (Carter et al., 2013; Petrou et al., 2018). However, as the idea of “working to live, not living to work” (Sturges and Guest, 2004) has permeated the modern workplace, interactions between team members have become increasingly important and diverse (Schlenkrich and Upfold, 2009; Hobman et al., 2004); indeed, many such exchanges arise not from formal job requirements nor are they explicitly task-oriented, yet they can positively shape work outcomes through various pathways (Tsui et al., 2000; Wang et al., 2008).

Based on this, we conceptualize Extra-Role Interpersonal Interaction (ERII) by its fundamental core—the behavioral domain of non-task-oriented, discretionary interactions occurring beyond the scope of formal job responsibilities, yet grounded in past or current work-role relationships. Although we believe this concept may occur in many cultural contexts, this study primarily explores it within the framework of Chinese *guanxi* culture. Thus, by focusing on ERII in Chinese organizations and teams, this research aims to clarify the concept and its structural dimensions, develop a measurement scale, and verify its validity, thereby constructing a theoretical foundation for discussing and explaining ERII’s impact. The scale developed in this study is designed specifically to map this core behavioral domain (i.e., what interactions occur). It is important to note that while these interactions are often characterized by high frequency, spontaneity, and contextuality in their natural manifestation, these attributes describe how the behaviors are typically enacted rather than define what they are. Our primary aim in this initial scale development is therefore to establish the foundational taxonomy of ERII behaviors. We posit that by mapping this behavioral domain, our research lays the groundwork for understanding how such commonplace, low-stakes interactions may cumulatively contribute to critical organizational outcomes, such as the spontaneous, cooperative behaviors encapsulated in Organizational Citizenship Behavior (Podsakoff et al., 2000).

## Extra-role interpersonal interaction and related constructs

### Theoretical foundations

The concept of a work role is central to organizational behavior. Murphy and Jackson (1999) define it as a set of performance responsibilities related to one’s position. Elaborating on this, Ashforth et al. (2000) conceptualizes role behavior as a manifestation of role identity within a social structure, while Sluss and Ashforth (2007) emphasize the purpose of roles is derived from their complementarity within embedded networks. In essence, organizational role theory posits that defining job functions and responsibilities is the cornerstone of discussing any organizational role.

Employees often enact a variety of roles, which are context-dependent and can be categorized by function, hierarchy, and status (Sluss and Ashforth, 2007). These encompass job-specific roles, relational roles (e.g., leader-subordinate), and broader identity roles as members of a department or the organization itself (Griffin et al., 2007; Ashforth et al., 2008). These multiple roles are nested within the organizational structure and can evolve in meaning and scope due to cultural, social, and situational factors, leading to role ambiguity and dynamism.

A fundamental distinction in this domain is between in-role and extra-role behavior. Katz and Kahn (1978) defined in-role behavior (IRB) as behaviors required to perform designated job duties, which are formally expected, evaluated, and rewarded by the organization (e.g., tasks in a job description; Becker and Kernan, 2003; Williams and Anderson, 1991). In contrast, extra-role behavior (ERB) refers to discretionary actions that benefit the organization but exceed prescribed role expectations (Van Dyne et al., 1994), and can be either positive or negative (Bolin and Heatherly, 2001).

This established theoretical dichotomy provides the foundational framework for our investigation. We now narrow our focus to a specific, under-examined domain within the broad spectrum of extra-role behavior: interpersonal interactions.

### Conceptual delineation and the cultural context of ERII

Building upon the theoretical foundation of extra-role behavior, we conceptualize Extra-role Interpersonal Interaction (ERII) as non-task-oriented, discretionary interactions that occur beyond the scope of formal job responsibilities, yet are grounded in past or current work-role relationships. To fully appreciate the prevalence and nuances of ERII, it is essential to consider the cultural context in which it is embedded.

Although the transformation of society by modern technologies has facilitated both the clashing and integration of diverse cultures, China’s local social and organizational culture nonetheless maintains an enduring anchor in the Confucian tradition. In most workplaces, interpersonal interaction among Chinese is a combination of multiple patterns of different sequences and the logic of action (Yum, 1988) and emphasizes harmony and resolution (Hwang, 1987; Leung et al., 2002). Chinese people tend to prefer to adopt diversified interaction practices with others in different relationships (Luo, 1997; Luo et al., 2016), reflecting the particularistic characteristics of both relationalism and a contract society (Chen et al., 2013). Two parties who are expected to have long-term contact or intersections tend to adopt attitudes to avoid conflicts, maintain face, control emotions, and create a harmonious atmosphere. In acquaintance relationships, instrumental behavior and emotional behavior are constantly and dynamically balanced to establish and expand connections (a self-centered trust network) to promote the sublimation of trust, interests, and other aspects (Luo, 2005; Luo, 2011). At the same time, the importance, understanding, perception, and expression of friendships between the two parties involved in the interactions are also significantly different from those in the West, especially in the sensitive perception of others’ friendships and the implicit expression of their own friendships.

Studies have found that in many Asian countries, including China, Korea and Japan, people are willing to form diverse, frequent, and informal social relationships (Oh et al., 2004; Horak and Klein, 2016; Horak et al., 2019; Zhang et al., 2005). In a workplace influenced by Chinese culture, new employees gradually acquire emotional expression norms adapted to the current organizational context through the observation, imitation, and exploration of interpersonal interaction patterns with their colleagues or superiors (Liu et al., 2015; Guo, 2019).

Compared with the Western social context, interpersonal interaction in the Chinese social and cultural background emphasizes long-term, stable, and harmonious interpersonal relationships (Flora Hung, 2004). As Liang (1990) stated, “Western people need to use wisdom, Chinese people need to use intuition–emotion […] The so-called discipline of filial piety and brother comity is everywhere still in love without me.” Similarly, Lin (1994) pointed out that “For Westerners, a viewpoint that is logically sound is often sufficient for acceptance. For the Chinese, however, logical correctness alone is insufficient; it must also be considerate of interpersonal harmony. In fact, this relational consideration often carries greater weight than pure logic.” This “human feeling (or *renqin*)” comprises the expression and expectations of emotions between people through reciprocal internal emotional care and external communication norms. Therefore, interpersonal interactions in Chinese society usually follow certain principles, including human preference, exchange, retention and appreciation, and the dynamic balance of human expression. These social norms have deeply penetrated all aspects of local culture, including local organizations and teamwork.

Therefore, the Chinese cultural context, with its emphasis on relational harmony (*guanxi*) and nuanced emotional expression (*renqing*), not only makes ERII a highly prevalent phenomenon but also imbues it with unique social and emotional significance that may differ from Western contexts.

### Discriminating ERII from related constructs

To further clarify the theoretical space of ERII, it is crucial to distinguish it from several adjacent yet distinct constructs in the literature. In existing studies, the concepts related to ERII mainly include social interaction, informal socializing ties, networking, informal interaction, and workplace friendship. Specific content reflected by these concepts includes micro-breaks, empathy.

First, it should be clarified that the ERII proposed in this study is a type of frequent interpersonal interaction based on past or current work role relationships that is not required by work norms. They may occur inside and outside the workplace and have a certain purpose; however, they may also be arbitrary. In contrast, social interaction includes formal and informal interactions and is a general interpersonal or intergroup communication activity and process (Ikeda et al., 2012). Informal social bonding refers to relationships with people who are willing to spend their free time with them (Mehra et al., 2001; Reagans and McEvily, 2003; Oh et al., 2004), the core of which lies in a state of connection rather than an interactive process. An interpersonal network is a dynamic psychological phenomenon that differs from static relationship structures. It is a behavioral network constructed for job performance, career development, and job searching (Porter and Woo, 2015a,b).

Informal and workplace friendships are more strongly associated with out-of-role interactions; however, there are clear differences between the two. Compared with formal interactions, informal interactions emphasize the attributes of transcending organizational structure, being non-procedural and non-coercive, and are part of social interaction. Informal interaction is generally defined as the construction or maintenance of interpersonal networks by organization members outside the workplace, work organization structure, or under the requirements of informal work (Liu et al., 2017; Pittaway et al., 2004; Winslow et al., 2019). The key difference between this and out-of-role human interaction is whether the interaction is a job or task requirement. Because there is a considerable proportion of informal interactions that undertake certain functions, such as performing relevant tasks for the organization, coordinating related people or teams, disseminating organizational culture or other information, and choosing indirect or informal communication channels, there is a certain degree of task-oriented in-role components. Workplace friendship is considered a type of informal, voluntary peer relationship in line with public norms and social–emotional goals based on work identity connections in organizations (Pillemer and Rothbard, 2018). This is the most intimate but unstable type of organizational peer relationship (Morrison, 2004). Workplace friendship is an evolved relationship state rather than a normal bond that emerges from many high-quality human interactions. There may be some similarities between the two in terms of expression, such as talking about shared interests with colleagues, joking around, or conversations about non-work topics. However, it is evident that ERII neither necessarily reflect relational closeness nor lead to the formation of close relationships. As Simmel (1949) observed, sometimes “talking is an end in itself.”

Furthermore, it is critical to distinguish ERII from the foundational Chinese cultural construct of *guanxi*. While both transcend formal roles, *guanxi* refers to a structural, enduring relational tie built on long-term reciprocity, obligation, and trust (Chen et al., 2013). In contrast, ERII constitutes the situated, behavioral processes (e.g., casual chats and shared leisure) through which such ties may be cultivated or maintained. This distinction positions *guanxi* as a relational outcome or structure, and ERII as the interactive, behavioral micro-foundation (Horak et al., 2019; Pillemer and Rothbard, 2018). This distinction underscores that *guanxi* is more structural and identity-based, while ERII is more processual and action-based. Together, they illustrate how Chinese workplace relations are simultaneously shaped by enduring cultural logics and everyday interactive practices.

In recent years, similar behaviors arising from work or job roles have received attention from different perspectives. For example, researchers have focused on micro-breaks and their positive effects on individuals or organizations and regard them as a strategy for energy recovery, physical and mental recovery, and relationship recovery, which can bring various benefits to individuals (Kim et al., 2018). Specifically, these behaviors may include browsing the web during work breaks, replenishing energy with snacks, relaxing or briefly disconnecting from the current state of work by chatting with colleagues about something other than work, and learning certain skills by reading short articles or videos in a fragmented time. Microbreaks are related activities that take advantage of work breaks, which may be related to colleagues or may simply be individual actions. The part that is associated with colleagues is often some non-task-oriented interpersonal interaction outside of work, which may occur in or outside of the workplace. This comprises the social dimension of micro-breaks in the workplace (Kim et al., 2018). Research on micro-breaks during work provides a broad understanding of the content of extra-role interpersonal interaction because the content and form of social interaction are not limited to information exchange or dinner parties. In contrast, people choose different methods and contents of interaction according to different situations and objects. These social interactions also reflect the individuals’ need to connect with others.

In addition, research has found that people can not only build their own social networks and interaction circles but also generate emotional resonance during interaction, which makes it easier for them to understand each other and empathize (Galinsky et al., 2008). In fact, there is a significant difference between empathy and emotional interaction because emotional interaction does not allow both parties to empathize or reach the same level of empathy, but empathy is necessarily caused by emotional interaction. However, these emotion-related interactions are not included in the connotations of formal or informal interactions. In addition to the broad interactive content, extra-role interpersonal interaction should at least include interactions related to emotion from the perspective of research in the field of leisure. Individuals in organizations should pay more attention to their physical and mental health, especially to personal development and its meaning, redesigning the field of individual leisure by improving themselves and their environment with a positive attitude and participating in leisure activities and interactions that go beyond their job responsibilities. They may participate in or organize volunteer activities to this end (Berg et al., 2010). Leisure crafting explains individuals’ participation in or arrangement of activities and interactions outside of work from this perspective, including leisure experiences involving individuals and colleagues, such as hunting together and participating in mindfulness activities. Leisure crafting also shows concern for the aspects of individuals in an organization that dissociate themselves from the content of their work and may relate to their work colleagues. Relevant studies have also confirmed the positive effects of leisure crafting involving colleagues on individuals and work (Table 1).

**Table 1:** The concepts related to ERII.

Concepts	Definition	Similarity to ERII	Distinction from ERII	What ERII is NOT
Social interaction	Interpersonal/group-based interactions enhancing support, commitment, and norms (Ross and Mirowsky, 1989; Cohen and Wills, 1985).	Conceptual overlap; involves inclusion dynamics	Broader scope: includes macro/micro levels, formal/informal forms	Broad-scope interaction beyond work roles
Interpersonal interaction	Building/maintaining networks inside/outside work, often informal (Liu et al., 2017; Pittaway et al., 2004; Winslow et al., 2019).	Content overlap (e.g., recreational activities)	Focus on informal systems; may include task-oriented supplement	Informality as defining trait; task-required contact
*guanxi*	Ties as strategies to gain competitive advantage, or as mechanisms of contracting and transactions (Park and Luo, 2001; Peng and Luo, 2000; Chen et al., 2013).	Shared social identities	Centered on the quality of ties and the structural, not focus on the actions or activities of interaction	Long-term obligatory bonds; instrumental exchange
Informal social bonding	Willingness to spend leisure time together (Mehra et al., 2001; Reagans and McEvily, 2003; Oh et al., 2004).	Non-task-oriented	Emphasizes connection state, not interaction process	A state of connectedness or bonding willingness
Networking	Behavior-driven network for work/career purposes (Porter and Woo, 2015a,b)	Dynamic, not just structural	Focus on relationship construction/state; not interaction process	Strategic, instrumentally-driven behavior
Workplace friendship	Informal, emotional peer relationships based on work identity (Morrison, 2004).	Similar expressions (e.g., non-work talks)	Reflects relationship intimacy/instability; erii is role-external	The outcome of a close affective relationship
Micro-breaks	Short, voluntary non-work activities (social, relaxation, cognitive, nutritional) (Kim et al., 2018).	Social dimension involves interaction	Not necessarily interpersonal; can be solitary or external to work	A break-time activity; solitary or external socializing
Empathy	Understanding others’ emotions through perspective-taking in interaction (Galinsky et al., 2008).	Involves emotional interaction and response	Arises from emotional exchange; erii includes broader behavioral forms	An internal cognitive-affective state
Leisure crafting	Purposeful redesign of leisure for development, meaning, health (Berg et al., 2010).	May involve social leisure activities	Focus on individual leisure activities; not necessarily interpersonal	Self-oriented design of personal leisure

Source: Authors’ own work.

In summary, while ERII has not been formally conceptualized, prior research has laid important epistemological groundwork. As Marshall (1986) observed, the boundary between work and leisure is often blurred, with “usually prudent work and play activities taking place simultaneously here [in the workplace].” Recent studies further highlight evolving workplace interactions, noting that increased interdependence and technological shifts have reshaped the ambiguity of roles and relationships among colleagues (Pillemer and Rothbard, 2018). Such changes facilitate the crossing of role boundaries through overlapping professional and social interactions (Yang and Wong, 2020). Researchers have also identified non-work domains beyond the family context, such as leisure spheres within organizational settings (Liang, 2018). Against this backdrop, ERII provides a nuanced and context-sensitive framework for capturing the broad range of low-obligation, daily interactions that constitute the informal fabric of organizational life. It represents the relational and behavioral micro-foundation upon which more formalized positive outcomes, including but not limited to OCB, may be built. By encompassing exchanges that are neither purely instrumental nor deeply emotional, ERII may fill a conceptual gap in the literature and offers a refined tool for examining how subtle, extra-role social processes influence workplace outcomes.

## Development and validation of the ERII scale

### Study 1: item development

#### Participants and procedure

This observational study was conducted following confirmation from the university Research Ethics Committee that no ethical approval was required. All participants provided voluntary informed consent prior to their involvement.

We conducted in-depth, semi-structured interviews with 48 working professionals from diverse organizational backgrounds in China. The interviews, which followed a predefined protocol (see Appendix A), spanned approximately 6 months, with each session lasting 30–40 min on average. The initial phase involved offline interviews with individuals from the researchers’ professional networks and employees from other organizations pertinent to the research themes. In addition to the structured questions, participants were encouraged to elaborate on their personal views regarding the role and significance of extra-role interpersonal interactions. All interviews were systematically documented and transcribed verbatim, yielding a textual corpus of 88,778 words that formed the foundation for subsequent coding and conceptual analysis.

The sample exhibited a balanced distribution in terms of gender (54.2% female, 45.8% male) and age, with the majority (77.1%) under 40 years old. Participants also varied in organizational tenure: 39.6% had less than 5 years, 31.2% had 5–10 years, and 29.2% had over 10 years of experience. They were recruited from state-owned (27.1%), private (35.4%), foreign-owned (18.8%) enterprises, and public institutions (18.7%), primarily based in Southwest (39.6%) and Southeast (33.3%) China.

#### Coding process

We analyzed the data following the coding procedures of classic grounded theory (Glaser and Strauss, 1967) using NVivo 20.0. The process unfolded in three stages: First, during open coding, we generated 307 initial nodes from the transcripts. Second, through axial coding, we condensed these into 36 coherent concepts. Finally, theoretical coding allowed us to abstract these concepts into 13 subcategories and the three core categories presented in Table 2. A detailed account of the node structure and analysis process is provided in Appendix A. Below is an example of encoding for the original text.

**Table 2:** Theoretical coding results of ERII.

Dimensions	Sub-dimensions		Nodes
BI	BI1	Relationship building and consolidation	Relationship building and needs
			Relationships maintaining or strengthened
	BI2	Acceptance and promotion	Expression of care
			Party and dine
	BI3	Companionate behavior and activities	Accompany with
			Companion behavior
			Travel and play together
	BI4	Identity or relationship identification	Team building activities
			Volunteer activities
			Follow and identify
RI	RI1	Recreational activities and leisure	Participate in cultural activities
			Meet for recreational activities
	RI2	Participatory and recreational activities	Chitchat
			Chess and card activities
			Fun experience
			Leisure experience
	RI3	Hobby club	Establishment interest club
			Interest activity participation
	RI4	Sports activities	Participate in sports activities
			Organizing sports activities
EI	EI1	Emotional connection	Comfort and show concern
			Express gratitude
			Listen and share
			In-depth communication
	EI2	Emotional building and strengthening	Kinship contact and gatherings
			Empathy pursuit
			Friendship building and maintenance
	EI3	Emotional release and relief	Relieve mood and stress
			Negative emotion catharsis
	EI4	Emotional information sharing	Share fun and feelings
			Share good news and thanks
	EI5	Supportive emotional expression	Bear with
			Celebration and cheerful activities
			Support and encourage each other
			Appreciative communication
			Gift exchange

BI, interaction for belongingness; RI, recreational interaction; EI, emotional interaction. This information was encoded in this study.

*Interviewee No. 39(original in Chinese): My leader, Manager Wang, and I both like outdoor hiking. Every month, we arrange a weekend hiking activity and invite colleagues to join us. During the walk, we help each other share stories, and after the walk, we enjoy a meal at a nearby farmer’s house. These spontaneous interactions allow us to relax after work, and also promote friendship and team cohesion between us. In addition, my colleague Xiao Li and I like basketball very much. We often play basketball in a nearby court after work. On the court, we are not only teammates but also competitors: we learn each other’s skills and play each other. After the game, we go to a nearby restaurant to have dinner and share interesting things that happened that day, which makes our relationship closer and makes work more enjoyable.*

The initial concept coding process for the original data provided by interviewee 39 is as follows: “My leader Manager Wang and I both like outdoor hiking, and we arrange a weekend hiking activity every month” and “My colleague Xiao Li and I like basketball very much, and we often play basketball in a nearby basketball court after work,” coded as “establishment interest club (RI31)”; “During the walk, we help each other share stories” is coded as “listen and share (EI13)”; “Enjoy a nice meal at a nearby farm” and “after the game we will go to a nearby restaurant for dinner” coded as “party and dine (BI21)”; “We learn from each other’s skills” is coded as “friendship building and maintenance (EI23)”; “Share the fun of the day” is coded as “share fun and feelings (EI41)”; and “These spontaneous interactions allow us to relax after work,” is coded as “relieve mood and stress (EI31).”

Following the classical rooted coding program, after forming the initial concept, the common parts of the 36 initial concepts were summarized to extract common factors and form subcategories (see Table 2). Furthermore, theoretical coding was performed to explain and summarize the subcategories.

The coding data and process resulted in extra-role interpersonal interactions in three dimensions: belonging-, leisure-, and emotional-type interactions. From the perspective of interactive content and behavioral motivation presented by coding, the content contained in these core categories is distinguishable from the existing relevant conceptual connotations.

#### Item development

Following the initial refinement of conceptual dimensions through coding, the study produced extensive descriptive data on ERII. In Study 1, items were developed based on the dimensions and categories derived from coding, thereby establishing a preliminary item pool for the ERII Scale. Guided by scale development principles proposed by Carpenter (2018) and Hinkin (2005), we carried out the item generation process.

Item development followed an inductive approach (Hinkin, 2005). From 36 identified nodes, an initial set of 36 questionnaire items was generated. Subsequently, two professors of human resource management and two experienced HR department heads evaluated the representativeness and dimensional coverage of these items. The experts provided independent ratings. Using the Delphi technique, four raters independently expressed agreement (“agree”) or disagreement (“disagree”) for each item. Items were retained only if at least 75% of the raters indicated agreement (Diamond et al., 2014).

In the first round, 22 items reached the 75% threshold, 10 items fell between 50 and 75%, and 4 items scored below 50%. In the second round, the four experts convened to discuss item wording and interpretation. Through consensus, redundant or unclear items were revised, merged, or removed. Following this process, the item set was reduced to 19. Independent evaluations confirmed that items with less than 75% agreement were discarded, while those above the threshold were refined through further expert feedback. Ultimately, a preliminary 19-item scale was finalized (see Table 3).

**Table 3:** Initial measurement items and dimensions of ERII.

Dimension	Item	
BI	Q1	Organize some social activities to promote friendship.
	Q2	Organize activities with people who share common interests.
	Q3	Organize and participate in relaxing outdoor activities.
	Q4	If available, take part in some volunteer activities
	Q5	Share personal updates or lighthearted moments to build rapport with colleagues.
	Q6	Chat to get to know each other better.
RI	Q7	Connect after work because of shared interests.
	Q8	Engage in some leisure activities in spare time after work.
	Q9	Take part in some recreational activities after work.
	Q10	Set up some hobby and interest groups after work.
	Q11	Organize informal social gatherings with colleagues (e.g., group outings).
	Q12	Talk about common topics or hobbies.
EI	Q13	Dine with colleagues with the specific aim of strengthening personal connections.
	Q14	Greet each other or send our best wishes on important holidays or birthdays.
	Q15	Connect over topics of mutual interest.
	Q16	Express concern to each other when we need to.
	Q17	Celebrate each other when work is done well or important progress is made.
	Q18	Complain when a task is not going well or we are in trouble.
	Q19	Talk about troubles in life and seek advice from each other.

BI, interaction for belongingness; RI, recreational interaction; EI, emotional interaction.

All interviews, coding, and initial item generation were conducted in Chinese. The items presented in this paper are professional translations of the original Chinese items. Throughout the scale development process, the assignment of items to their theoretical dimensions (BI, RI, and EI) remained consistent with the original qualitative coding structure.

### Study 2: exploratory factor analysis

#### Sample and procedure

Data collection for pre-test analysis was conducted through online questionnaires in China. Participants were working adults recruited through two primary channels: (a) the online platform Credamo, and (b) referrals from colleagues and alumni networks. To ensure data quality, we implemented a screening protocol prior to analysis. Responses were excluded if they were (1) excessively rapid (completion time below two standard deviations from the mean), (2) perfunctory (e.g., exhibiting straight-lining patterns or providing nonsensical text in open-ended questions), or (3) logically inconsistent (e.g., failing embedded attention check items). After applying these rigorous cleaning criteria, 359 valid responses were retained for analysis, resulting in an effective recovery rate of 94.474%.

According to the statistics, the pre-test subjects were 359 members of organizations from Southwest and Southeast China. Approximately 61.3% of the participants were female, 41.5% were under the age of 30, 36.5% had organizational tenure of less than 5 years, and the type of organization was diversified.

#### Measures

The data for Study 2 were collected using an ERII questionnaire consisting of 19 items (Table 3). A 5-point Likert scale ranging from 1 (“completely disagree”) to 5 (“completely agree”) was used for measurement and testing.

#### Results

Statistical software (SPSS 23.0) was used to analyze the pretest data. The absolute value of the skewness coefficient and the absolute value of the kurtosis coefficient of the 19 initial items of the initial scale of extra-role interpersonal interaction were less than 3. The absolute value of the kurtosis coefficient was less than the critical value of 10, and the sample normality test passed.

First, we applied a minimum factor loading threshold of 0.40 to determine whether an item should be retained in the scale (Churchill, 1979). Items were eliminated based on two criteria: (1) those with factor loadings below 0.40 on all factors (indicating a weak relationship with the latent construct), and (2) those that loaded more highly on a factor other than the one for which they were theoretically intended (indicating a lack of discriminant validity). This process resulted in the removal of six items. Second, we conducted an exploratory factor analysis (EFA) to examine the factor structure and psychometric properties of the remaining 13 items. The Kaiser–Meyer–Olkin (KMO) measure of sampling adequacy and Bartlett’s test of sphericity confirmed the suitability of the data for factor analysis (Worthington and Whittaker, 2006). Principal component analysis with varimax rotation was then performed. While the factors of belonging, recreational, and emotional interaction were theoretically expected to correlate, which was later confirmed by the confirmatory factor analysis in Study 3, we initially employed the varimax rotation for a more conservative and parsimonious initial extraction of factors. This approach is sometimes adopted in early scale development to obtain a clear, orthogonal solution for initial item screening and structure exploration (Worthington and Whittaker, 2006). Factor retention was evaluated using the eigenvalue-greater-than-one rule, the scree test, and parallel analysis (Fabrigar and Wegener, 2012). This procedure supported a three-factor solution, which accounted for 35.326, 8.810, and 7.839% of the variance, explaining a total of 51.974%. Third, only items with strong loadings on their designated factor and weak cross-loadings were retained (Zhang et al., 2011). Based on this criterion, item Q19 was removed. Although talking about troubles involves emotion, the point that seeking advice was also deemed more instrumental and relationally consolidating, aligning it more closely with the conceptual boundary of Belonging Interaction. However, as it loaded ambiguously, it was excluded to enhance the discriminant validity of the final scale. Following these steps, the final 12-item ERII scale, along with factor loadings, is presented in Table 4.

**Table 4:** Exploratory factor analysis for ERII.

Measurement items		Factor loading		
		1	2	3
Q1	Organize some social activities to promote friendship.	0.600		
Q2	Organize activities with people who share common interests.	0.671		
Q3	Organize and participate in relaxing outdoor activities.	0.675		
Q4	If available, take part in some volunteer activities.	0.689		
Q7	Connect after work because of shared interests.		0.588	
Q8	Engage in some leisure activities in spare time after work.		0.636	
Q9	Take part in some recreational activities after work.		0.634	
Q10	Set up some hobby and interest groups after work.		0.641	
Q11	Organize informal social gatherings with colleagues (e.g., group outings).		0.595	
Q13	Dine with colleagues with the specific aim of strengthening personal connections.			0.587
Q14	Greet each other or send our best wishes on important holidays or birthdays.			0.646
Q15	Connect over topics of mutual interest.			0.685
Q19	Talk about troubles in life and seek advice from each other.	0.529		

*N* = 359. Factor analysis was based on principal component analysis and varimax rotation. The original items were translated from Chinese.

### Study 3: confirmatory factor analysis

#### Sample and procedure

Questionnaire collection was conducted online in China. An online questionnaire was distributed to 760 working adults through alumni and professional acquaintance networks. Following data collection, we applied the same data cleaning procedure as in Study 2, which involved removing responses that were excessively rapid, perfunctory, or logically inconsistent. This process yielded 715 valid questionnaires, with an effective recovery rate of 94.079%.

According to the statistics, the pre-test subjects were 715 members of organizations from Southwest and Southeast China. Among the participants, approximately 59.3% were female, 40.8% were under the age of 30, 36.5% had organizational tenure of less than 5 years, and the type of organization was diversified.

#### Measures

The data for Study 3 were collected using an ERII questionnaire consisting of 12 items (see Table 4). A 5-point Likert scale ranging from 1 (“completely disagree”) to 5 (“completely agree”) was used for measurement and testing.

#### Results

Two reliability indicators were analyzed in this round. First, the overall internal consistency reliability of the scale was analyzed. Second, the correlation coefficient between each item and the overall questionnaire was calculated (Koo and Li, 2016). These two analyses were mainly used to test the statistical consistency and relevance of each item on the formal scale, which was the premise for further verification of the scale dimension construction.

According to the internal consistency reliability analysis, the overall Cronbach’s *α* coefficient of the ERII questionnaire was 0.840, indicating good stability and consistency of the questionnaire items. The Cronbach’s α coefficients corresponding to interaction for belonging, recreational interaction, and emotional interaction were 0.751, 0.761 and 0.835, respectively, indicating that the stability and consistency of items in each dimension were acceptable. Additionally, the correlation coefficient CITC of the overall questionnaire for each item was greater than 0.4. All 12 items passed the reliability test.

Amos 28.0 was used to construct a structural equation model, and the convergence validity of each dimension was reflected through the estimation and fitting test of the measurement model. Model estimation and fitting focus on non-standardized regression coefficients, standardized regression coefficients, composite correlation coefficients, standard errors, critical ratios, combined reliabilities, and mean variance extraction values (Fornell and Larcker, 1981; Hair et al., 2009).

From the path coefficient analysis of the measurement model, the standardized regression coefficients of the four items of interaction for belonging were between 0.622–0.776 (>0.6), indicating good convergent validity (Hair et al., 2009). In addition, judging from the fitting index of the latent variable model, the critical ratios of Q2, Q3, and Q4 were C. R. These were 14.876, 14.643, and 15.799, respectively, with a significance level of 0.001. A combined reliability of CR = 0.771 greater than 0.7, average variance extraction value AVE = 0.458.

From the path coefficient analysis of the measurement model, it can be seen that the standardized regression coefficients of the five items of recreational interaction are between 0.517–0.832, (>0.5), which can be considered to have acceptable convergence validity. In addition, judging from the fitting index of the latent variable model, the critical ratios of Q8, Q9, Q10, and Q11 is C. R. These were 13.265, 16.034, 13.611, and 17.177, respectively, with a significance level of 0.001. The combined reliability of CR = 0.780 was greater than 0.7, and the average variance extraction value was AVE = 0.421.

From the path coefficient analysis of the measurement model, it can be seen that the standardized regression coefficients of the three items of affective interaction are between 0.697 and 0.928, greater than 0.6, which can be considered to have acceptable convergence validity. In addition, judging from the fitting index of the latent variable model, the critical ratios of Q14 and Q15 were C. R. These values are 21.732 and 19.365, respectively, with a significance level of 0.001. The component reliability, CR = 0.844, is greater than 0.7, and the mean variance extraction value, AVE = 0.644, is greater than 0.5.

In summary, the three dimensions of extra-role interpersonal interaction – interaction for belonging, recreational interaction, and emotional interaction – demonstrate strong internal consistency and provide initial evidence for the convergent validity of the measurement model, as indicated by the significant factor loadings and composite reliabilities. Further evidence for the scale’s convergent validity with external criteria is established in Study 4.

Based on the actual situation, five competitive models are included in the analysis in this section. Five latent variable models were plotted separately in AMOS 28.0, and model-fitting estimates were performed. According to the suggestions of Heckman and Walker (1987), the fitting of the five competing models is shown in the table below. According to the principle of fit reduction and rationality, M1 model is the best three-dimensional model of extra-role interpersonal interactions (see Table 5).

**Table 5:** Confirmatory factor analysis for ERII.

Models	𝜒^2^	𝑑𝑓	Δ𝜒^2^(𝑑𝑓)	𝜒^2^/𝑑𝑓	TLI	CFI	AGFI	RMSEA
M1	331.918	51		6.508	0.882	0.909	0.901	0.088
M2	575.582	53	243.664(2) ***	10.860	0.789	0.831	0.820	0.118
M3	933.795	53	601.877(2) ***	17.619	0.645	0.715	0.676	0.153
M4	936.295	53	604.377(2) ***	17.666	0.644	0.714	0.710	0.153
M5	1174.675	54	842.757(3) ***	21.753	0.557	0.637	0.669	0.170

***p < 0.001. *N* = 715. M1 = BI, RI, EI; M2 = BI + RI, EI; M3 = BI + EI, RI; M4 = RI + EI, BI; M5 = BI + RI + EI.

The standardized factor load of the 12 items of the three-factor model in their respective dimensions ranges from 0.53 to 0.88, indicating that the basic fit of the three-factor model is ideal, and that the three-dimensional structure of extra-role interpersonal interaction is supported by statistical tests.

### Study 4: validity analysis

#### Sample and procedure

Participants for this study were invited to complete an online survey in China. We distributed 240 questionnaires to working adults recruited via professional networks. When responding to the ERII scale, participants were instructed to reflect on their typical patterns of interaction with colleagues beyond formal job duties. This framing implicitly captures behaviors that are recurrent and integral to their regular interpersonal landscape, which aligns with the conceptual attributes of frequency and spontaneity.

After collecting the sample data, we performed a preliminary screening using the same criteria established in Studies 2 and 3 (removing excessively rapid, perfunctory, or logically inconsistent responses). This resulted in 228 valid questionnaires, with an effective recovery rate of 95%.

According to the statistics, the pre-test subjects were 228 members of organizations from Southwest and Southeast China. Among the participants, approximately 64.5% were female, 50.9% were under the age of 30, 43.4% had organizational tenure of less than 5 years, and the type of organization was diversified.

#### Measures

As *informal interactions* overlap with extra-role interpersonal interactions in content, such as entertainment and leisure activities under non-work requirements and personalized social activities, we first chose to compare informal interactions with extra-role interpersonal interactions. The informal interactions used in this section were the behavioral dimension of the informal interaction scale in the workplace compiled by Winslow et al. (2019), with a total of six items. Examples include “My colleagues and I talk about shared interests,” “I often talk about non-work related topics,” and “I discuss with my colleagues what happens during my non-work hours (such as weekends or evenings).”

In addition, *workplace friendships* and extraterritorial interactions were found to be similar in terms of expression, with the most relevant components being high-quality, belonging-oriented interactions or relationship building. Based on this, this section adopts the workplace friendship intensity scale from the Workplace Friendship Scale compiled by Nielsen et al. (2000), focusing on a comparative analysis of extra-role interpersonal interaction and its belonging-type interaction dimensions. There are four questions on the strength of workplace friendships, including “I feel I can trust many colleagues” and “I have formed strong friendships at work.”

*Social activities* during micro-breaks are considered activities of communication between individuals and colleagues, family members, or friends during work breaks, which may be related to ERII and leisure interactions. This section uses the social activity dimension of the micro-break scale compiled by Kim et al. (2018) for comparison and testing. The social activity part consists of three items, including “I will talk with my colleagues about non-work related topics,” “I will send text messages or call my friends or family members during breaks from work,” and “I will check my personal social media accounts or circle of friends during breaks from work.”

*Empathy* is considered to be related to emotional interactions, which may in turn be related to extra-role interpersonal and affective interactions. Therefore, the positive empathy part of the Positive and Negative Empathy Scale compiled by Andreychik and Lewis (2017) was tested and analyzed in this section. The Positive Empathy Scale consists of seven items, including “I feel uplifted when people around me are in high spirits,” “I feel happy when people around me are laughing,” and “I feel happy when people receive gifts.”

We adopted Brislin’s (1980) translation and back-translation procedures to generate a Chinese version of the measures. All scales were measured using a 5-point Likert scale, where 1 = strongly disagree and 5 = strongly agree.

#### Results

##### Correlation analysis

Criterion validity is typically used to evaluate the degree of correlation between a scale and related constructs, serving as a key reference for assessing the validity of scale development. In this study, Pearson’s correlation coefficient was used to examine the relationships between extra-role interpersonal interaction and several criterion scales (informal interaction, workplace friendship, micro-breaks, and empathy). A correlation coefficient between 0.4 and 0.8 is generally considered acceptable (see Table 6). Additionally, since the data for the five constructs were collected from the same sample, we conducted Harman’s single-factor test to assess potential common method bias. The results showed that a single factor accounted for 32.002% of the total variance, which is below the recommended threshold of 50%, indicating that common method bias is not a serious concern in this study.

**Table 6:** The correlation coefficient between ERII and related scales.

Variable/dimensions	Informal interaction	Workplace friendship	Micro-breaks	Empathy
ERII	0.463^**^	0.768^**^	0.056	0.706^**^
BI	0.412^**^	0.695^**^	0.035	0.661^**^
RI	0.434^**^	0.722^**^	0.043	0.664^**^
EI	0.415^**^	0.671^**^	0.080	0.590^**^

**p < 0.01. ERII, extra-role interpersonal interaction; BI, interaction for belongingness; RI, recreational interaction; EI, emotional interaction.

Regarding the correlation coefficient, except for microbreaks at work (*r* = 0.056, *p* > 0.01), ERII was significantly correlated with informal interaction, workplace friendship, and empathy, indicating that ERII was partially correlated with these three constructs. The correlation coefficients between ERII and workplace friendships (*r* = 0.768, *p* < 0.01) and empathy (*r* = 0.706, *p* < 0.01) were higher than those between ERII and informal interactions (*r* = 0.463, *p* < 0.01). It was further tested that ERII is not only related to informal interaction but also to informal interaction in the connotation and emphasis of constructs, but also has the meaning of connecting with specific groups.

##### Convergent and discriminant validity analysis

Convergent validity, which indicates the extent to which indicators of a specific construct converge or share a high proportion of common variance, is typically judged by two criteria: the Average Variance Extracted (AVE) and Composite Reliability (CR). Conventionally, an AVE value greater than 0.5 and a CR value greater than 0.7 are considered indicative of adequate convergent validity (Fornell and Larcker, 1981; Hair et al., 2009). Our data indicate that, although the AVE values for the informal interaction and empathy constructs were slightly below the stringent threshold of 0.5, their respective CR values were all above 0.6 (see Table 7), exceeding the more liberal threshold for exploratory research as noted in the literature (e.g., Nunnally and Bernstein, 1994). Given that CR is a more robust indicator of internal consistency reliability, and considering the exploratory nature of this study, the convergent validity of these constructs was deemed acceptable Hair et al. (2009).

**Table 7:** Discriminant validity of ERII and criterion scale.

Variable/dimensions	Informal interaction	Workplace friendship	Micro-breaks	Empathy
Informal interaction	**0.540**			
ERII	0.463**			
Workplace friendship	0.477^**^	**0.713**		
BI	0.412^**^	0.695^**^		
Micro-breaks	0.255^**^	0.041	**0.750**	
RI	0.434^**^	0.722^**^	**0.043**	
Empathy	0.481^**^	0.745^**^	0.034	**0.626**
EI	0.415^**^	0.671^**^	0.080	0.590**
AVE	0.292	0.508	0.562	0.392
CR	0.674	0.804	0.791	0.817

**p < 0.01. ERII, extra-role interpersonal interaction; BI, interaction for belongingness; RI, recreational interaction; EI, emotional interaction; CR, composite reliability. The underscored and bold value represents the square root of the corresponding latent variable, AVE.

Discriminant validity is an important means of testing the degree of difference between latent variables (Henseler et al., 2015), and the AVE method is commonly used. The AVE method first builds measurement models of different latent variables, calculates their standardized regression coefficients, calculates the AVE value of each latent variable, and compares the square root of the AVE value with the correlation coefficient of the corresponding variable. If the AVE square root value of a latent variable is greater than the correlation coefficient with other variables, it is considered to have good discriminant validity (Fornell and Larcker, 1981).

According to the discriminant validity test, the AVE square root of informal interaction 0.540 is greater than the correlation coefficient between informal interaction and character interpersonal interaction (0.463) and is also greater than the correlation coefficient of other variables and dimensions. The AVE square root of workplace friendships (0.713) was greater than the correlation coefficient of interaction (0.695). The AVE square root of microbreaks was much higher than the correlation coefficient of recreational interaction (0.043). The AVE square root of empathy (0.626) was larger than the correlation coefficient of emotional interaction (0.590). The results of discriminant validity of ERII and criterion scale are in Table 7.

##### Preliminary incremental validity analysis

We performed a hierarchical regression analysis to preliminarily explore whether ERII explains additional variance in workplace friendship beyond that accounted for by informal interaction. As shown in Table 8, M1, which included only informal interaction, explained a significant 30.9% of the variance in workplace friendship (*R*^2^ = 0.309). Importantly, when ERII was added in M2, it resulted in a significant increase in explained variance (Δ*R*^2^ = 0.367, *p* < 0.001). The total variance explained rose to 67.6%. This suggests that ERII captures a unique aspect of interpersonal dynamics beyond informal interaction, providing preliminary support for its incremental validity and highlighting its potential value as a distinct construct.

**Table 8:** Preliminary incremental validity analysis of ERII on WF (*N* = 228).

Variables	M1	M2
Constant	1.703***	0.457*
Informal interaction	0.586***	0.253***
ERII		0.628***
*R^2^*	0.309	0.676
Δ*R^2^*		0.367***
*F*	101.254***	234.736***

*p < 0.05, ***p < 0 0.001. ERII, extra-role interpersonal interaction; WF, workplace friendship.

In summary, ERII is correlated with informal interaction, workplace friendship, and empathy, but the correlation test is not significant for microbreaks between work hours. Judging from the difference in the content of the scale, ERII is distinguishable from the scales of these variables, which further proves the validity and discriminability of the ERII construct and the measurement.

## Discussion

Our multi-study-scale development process successfully delineates and validates the construct of ERII within Chinese organizational contexts. The findings across four studies consistently support ERII as a three-dimensional construct encompassing interaction for belonging, recreational interaction, and emotional interaction, measured by a reliable and valid 12-item scale.

This research refines the understanding of non-task-oriented interactions. Unlike the more static, obligation-laden strong ties characterized by *guanxi* (Chen et al., 2013), ERII captures dynamic, low-obligation, quotidian interactive behaviors that are grounded in work roles yet operate beyond formal duties. As revealed through both qualitative interviews and quantitative analysis, it is through seemingly minor acts—such as sharing meals, organizing interest-based activities, or expressing personal concerns that employees continuously build and maintain the social fabric of the workplace. Critically, the developed scale successfully discriminates ERII’s core behavioral domain from related yet distinct constructs. The correlation and discriminant validity tests confirm that while ERII is moderately to strongly correlated with workplace friendship and empathy, suggesting it may provide fertile ground for deeper relational bonds, it is not synonymous with these relational states. ERII emphasizes the process and act of interaction itself, aligning with Simmel’s (1949) notion that sometimes “talking is an end in itself.” The non-significant correlation with micro-breaks further underscores ERII’s specificity, focusing on substantive interpersonal engagement rather than any form of non-work activity. In addition, the incremental validity analysis demonstrates that ERII explains unique variance in workplace friendship beyond that accounted for by informal interaction. It should be noted that this analysis serves as a preliminary exploration of ERII’s predictive utility rather than a definitive test of criterion-related validity, which would require validation against more distal organizational outcomes (e.g., OCB). This confirms that ERII captures a distinct facet of workplace sociability not fully represented by existing constructs, solidifying its value as an independent variable for future research.

### Theoretical implications

In summary, this study makes several key theoretical contributions. First, it systematically conceptualizes and operationalizes ERII in the Chinese context, addressing a significant gap in the literature concerning the definition and measurement of routine, work-role-based yet non-instrumental interpersonal interactions. The grounded theory approach identifies three core behavioral dimensions, illuminating how organizational members initiate or participate in such interactions by adjusting or transcending their formal role perceptions.

Second, this study positions ERII as a critical behavioral precursor and relational conduit for OCB. While traditional OCB research focuses on discrete, beneficial acts such as helping or courtesy (Podsakoff et al., 2000; Ocampo et al., 2018; Goel and Singh, 2024), our framework elucidates the relational substrate from which such behaviors emerge. The three dimensions of ERII represent recurring, low-stakes interactions that build social capital and psychological safety (Aselage and Eisenberger, 2003). Activities like dining together (BI), engaging in sports (RI), or offering mutual support (EI) cultivate the socio-emotional conditions that make voluntary acts of organizational citizenship more likely and spontaneous. Thus, ERII provides a more granular mechanism explaining how informal social processes underpin formal organizational functioning (Cropanzano et al., 2017; Dierdorff and Rubin, 2022).

Third, the study clarifies ERII’s conceptual distinctiveness and cultural boundaries. While the three-dimensional structure may generalize to other collectivist cultures (e.g., Korea, Japan), its specific manifestations and drivers are likely uniquely shaped by Chinese cultural rules of *renqing* and *face* (Chang, 2012; Hwang, 1987; Zhang et al., 2005). Furthermore, ERII is conceptually distinct from other informal interactions aimed primarily at information exchange or networking, due to its fundamental lack of a task or in-role orientation.

### Practical implications

Our findings yield several actionable implications. First, organizations should formally recognize the value of ERII and foster its positive forms. Instead of mandated, scripted team-building, managers should facilitate organic, interest-driven micro-communities (e.g., hobby clubs, volunteer groups). Providing resources for such self-organized activities promotes authentic relationship-building while respecting employee autonomy.

Second, managers should design interaction-conducive environments and schedules. This includes creating informal shared spaces (e.g., lounges) and protecting time for non-task interaction (e.g., open-agenda team lunches). These structural adjustments lower barriers to spontaneous ERII, enhancing team cohesion and psychological safety.

Third, it is crucial to strengthen merit-based HR systems to mitigate potential risks like favoritism. Transparent performance criteria, team rotation, and clear ethical guidelines can help decouple interpersonal affinity from professional decisions, ensuring ERII enhances rather than undermines organizational fairness.

Finally, within the Chinese cultural context, leaders can consciously leverage ERII as a cultural resource to improve team stability and talent retention. By responsibly facilitating belongingness-, recreation-, and emotion-based interactions, organizations can build a more engaged, collaborative, and resilient workforce.

### Limitations and future research directions

While this research provides a validated scale and establishes the foundational nomological network for ERII, certain aspects present opportunities for further refinement and verification in future studies.

First, although we implemented procedural and statistical remedies to mitigate common method bias, we acknowledge that this approach has to some extant inherent limitations. To build a more robust and causally nuanced understanding of ERII, future research would benefit from employing longitudinal or experimental designs. For instance, tracking how fluctuations in ERII predict subsequent changes in team cohesion or OCB over time would significantly strengthen inferences about its developmental impact. Additionally, collecting multi-source data (e.g., pairing employee self-reports of ERII with peer-ratings of their OCB) would further bolster the validity of the findings by reducing same-source bias. To more rigorously establish the predictive and incremental validity of the measurement and construct of ERII, future studies should explicitly test its ability to explain variance in key organizational outcomes, such as OCB or team performance, ideally using multi-source or multi-wave data.

Second, the dynamic nature of ERII, characterized by attributes like spontaneity and contextuality was integral to our conceptualization but not directly captured by the reflective scale items. Our scale successfully maps the content of these interactions, establishing a crucial taxonomic foundation. A logical and valuable next step would be to employ high-fidelity methodological approaches such as Experience Sampling Methodology or diary studies. These methods are ideally suited to capture these interactions and allowing researchers to model the within-person antecedents and consequences of ERII.

Finally, concerning cross-cultural applicability, this study was contextualized within Chinese *guanxi* culture. The strong psychometric properties demonstrated here confirm the scale’s validity within this specific context. However, to establish its generalizability and explore potential cultural moderators, rigorous cross-cultural validation is essential. Future work should involve systematic translation and back-translation procedures, followed by tests of measurement invariance across diverse cultural samples. This would enable meaningful cross-cultural comparisons of how ERII functions globally.

## Conclusion

This study establishes Extra-role Interpersonal Interaction as a multifaceted construct vital to understanding the informal social landscape of Chinese organizations. Through a systematic, multi-method investigation, we developed and validated a three-dimensional (Belonging, Recreational, and Emotional) model of ERII, supported by a reliable and valid 12-item scale. The demonstration of ERII’s discriminant and incremental validity underscores its unique position in the nomological network of workplace constructs. Ultimately, this work peels back the “ineffable wonderful gauze” surrounding these pervasive interactions, establishing a solid foundation for their formal study and thoughtful management. A deeper understanding and strategic facilitation of ERII hold significant potential for enhancing organizational performance, employee well-being, and team resilience in an evolving workplace.

## Data Availability

The original contributions presented in the study are included in the article/Supplementary material, further inquiries can be directed to the corresponding author.
