# Supplementary material for: Extra-role interpersonal interactions in Chinese organizational contexts: construct, measurement, and validity
Source: Front Psychol. 2026 Jan 9;16:1597133. doi: 10.3389/fpsyg.2025.1597133 (PMC12827159; doi:10.3389/fpsyg.2025.1597133)
Supplement: Supplementary file 1 [file Supplementary_file_1.docx]

# Appendix A: Detailed Methodology for Study 1

**1. Participant Demographics in Study 1**

**Table B1 Detailed Participant Demographics (N=48)**

| **Characteristic** | **Category** | **Percentage (%)** |
| --- | --- | --- |
| **Gender** | Male | 45.8 |
|  | Female | 54.2 |
| **Age** | Under 30 | 41.7 |
|  | 30-40 | 35.4 |
|  | Over 40 | 22.9 |
| **Organizational Tenure** | < 5 years | 39.6 |
|  | 5-10 years | 31.2 |
|  | > 10 years | 29.2 |
| **Organization Type** | State-owned Enterprise | 27.1 |
|  | Private Firm | 35.4 |
|  | Foreign-owned Enterprise | 18.8 |
|  | Other (e.g., Public Institution) | 18.7 |
| **Region** | Southwest China | 39.6 |
|  | Southeast China | 33.3 |
|  | Northeast China | 10.4 |
|  | Other | 16.7 |

**2. Semi-Structured Interview Protocol**

2.1 Introduction and informed consent.

2.2 Demographic information collection.

2.3 Core Questions, including as follows:

1) At the workplace, what methods do you usually use to interact with colleagues or leaders? Please give 1-2 examples of spontaneous interpersonal interactions between you and your colleagues (or leaders).

2) Besides the examples you just gave, what else do you consider to be interpersonal interactions beyond job requirements? Please list as many as you can, and elaborate on 1-2 that interest you the most.

3) How do you view the phenomenon of interpersonal interactions with colleagues (or leaders) that are not driven by job requirements but still exist?

4) Under what circumstances would you participate in such interpersonal interactions?

5) After having supportive interactions with colleagues (or leaders), such as expressing concern, solving difficulties, or exchanging information, how do you feel? Why do you feel that way?

6) What positive impact do you think interactions with colleagues (or leaders) beyond tasks have on your work state or feelings?

7) Do you believe that more interactions with colleagues (or leaders) promote certain aspects of your work? In what ways?

Debriefing and thanks.

**3. Detailed Use of NVivo**

The qualitative data analysis followed a structured process within NVivo 20.0:

1) Data Import: All 48 transcribed interviews were imported into an NVivo project.

2) Open Coding: The researchers performed line-by-line coding, creating free nodes for each identified idea or action. This resulted in 307 raw, initial nodes.

3) Selective Coding: Using NVivo’s grouping and merging functions, the 307 free nodes were analyzed for thematic similarities. Related nodes were clustered into tree nodes to form the 36 initial concepts and then the 13 subcategories.

4) Theoretical Coding: The 13 subcategories (tree nodes) were further abstracted into the three core categories (dimensions) by examining their theoretical relationships and properties within NVivo. The software’s query and model visualization features were used to explore connections between categories and ensure they comprehensively covered the data.
